# Spatial and temporal distribution of phase slips in Josephson junction chains

**DOI:** 10.1038/s41598-017-11670-7

**Published:** 2017-09-13

**Authors:** Adem Ergül, Thomas Weißl, Jan Johansson, Jack Lidmar, David B. Haviland

**Affiliations:** 1 Nanostructure Physics, Royal Institute of Technology, SE-106 91 Stockholm, Sweden; 2 Department of Natural Sciences, University of Agder, Kristiansand, Norway; 3 Theoretical Physics, Royal Institute of Technology, SE-106 91 Stockholm, Sweden; 4 Present Address: Department of Physics, Stockholm University, SE-106 91 Stockholm, Sweden

## Abstract

The Josephson effect, tunnelling of a supercurrent through a thin insulator layer between two superconducting islands, is a phenomena characterized by a spatially distributed phase of the superconducting condensate. In recent years, there has been a growing focus on Josephson junction devices particularly for the applications of quantum metrology and superconducting qubits. In this study, we report the development of Josephson junction circuit formed by serially connecting many Superconducting Quantum Interference Devices, SQUIDs. We present experimental measurements as well as numerical simulations of a phase-slip center, a SQUID with weaker junctions, embedded in a Josephson junction chain. The DC transport properties of the chain are the result of phase slips which we simulate using a classical model that includes linear external damping, terminating impedance, as well as internal nonlinear quasiparticle damping. We find good agreement between the simulated and the experimental current voltage characteristics. The simulations allow us to examine the spatial and temporal distribution of phase-slip events occurring across the chains and also the existence of travelling voltage pulses which reflect at the chain edges.

## Introduction

Josephson junction chains (JJCs) are a model system for understanding superconductivity in one dimension^1^. They find important technological application in quantum metrology for realization of the SI unit Volt^2^ and as a microwave amplifier for quantum-limited signal amplification^3, 4^. In these examples the condensate phase is considered as a classical variable, with nonlinear dynamics that is essential to the device function. There have also been several experiments demonstrating quantum dynamics of the phase in JJCs, such as the superconducting-to-insulator quantum phase transition and the Coulomb blockade of Cooper pair tunneling^5–13^. Quantum phase slips (QPS) localized to one point in a circular JJC have been exploited for a promising new type of superconducting qubit called the Fluxonium^14, 15^.

The JJC is characterized by a spatially distributed phase of the superconducting condensate *θ*(*x*, *t*). The low-temperature dynamics of this phase depends on three characteristic parameters of the individual junctions: The Josephson Coupling energy *E*_*J*_ = *I*_*C*_ħ/2*e*, where *I*_*C*_ is the junction critical current; the single electron charging energy *E*_*C*_ = *e* ^2^/2*C*, where *C* is the junction capacitance; and the damping in the electromagnetic environment seen by the junction, represented by a series resistance *R*_damp_. The dynamics can be described in terms of the spatial and temporal distribution of phase slip events, or the winding by an amount 2*π*, of the phase difference between nearest neighbours. Phase slips can be triggered by thermal activation^16, 17^, by quantum tunneling^18–21^, or simply by applying a large enough bias in the presence of strong enough damping. A voltage bias fixes the external winding rate of the phase at the edge of the array, ${V}_{{\rm{bias}}}\propto \dot{\theta }\,\mathrm{(0},\,t)-\dot{\theta }\,(L,\,t)$, and the damping may be the result of quasi-particle tunneling in the individual junctions, or radiation damping resulting from the excitation of linear modes of the JJC.

It is useful to classify the dynamics in terms of two dimensionless ratios involving these three characteristic parameters: *E*_*J*_/*E*_*C*_, and *R*_damp_/*R*_*Q*_, where *R*_*Q*_ = *h*/4*e* ^2^ = 6.45 *k*Ω is the quantum resistance. These numbers determine whether the condensate ground state is best described in terms of classical phase (i.e. large quantum fluctuations of the number of Cooper pairs) or in terms of a quantum phase (i.e. classical, well defined number of Cooper pairs). In a voltage biased configuration, classical behavior of the phase is promoted when ${E}_{J}/{E}_{C}\gg 1$, and quantum behavior of the phase when ${E}_{J}/{E}_{C}\ll 1$. However, in the interesting regime where ${E}_{J}/{E}_{C}\simeq 1$, it is the damping which plays the decisive role. When ${R}_{Q}/{R}_{{\rm{damp}}}\gg 1$, quantum phase-slips are rare and the JJC can be described as a Josephson element with a modified current-phase relation^22^. When ${E}_{J}/{E}_{C}\simeq 1$ and ${R}_{Q}/{R}_{{\rm{damp}}}\ll 1$ quantum phase slips dominate and the dynamics is more easily described in terms of ‘charge-slips’, or the passage of single quanta of charge.

The role of the damping resistance *R*_damp_ is particularly interesting in long JJC’s because the linear dynamics of the chain is that of a high impedance transmission line for distributed Josephson plasmon modes^23^. In an infinite array (or finite array with matched termination), well below the plasma frequency of the individual junctions $\omega \ll {\omega }_{p}=2\pi {f}_{p}=\sqrt{8{E}_{J}{E}_{C}}/\hslash$ we find a linear dispersion relation describing a frequency-independent characteristic impedance ${Z}_{C}=({R}_{Q}/\pi )\,\sqrt{2{E}_{C0}/{E}_{J}}$ ^24–27^. *E*_*C*0_ = *e* ^2^/2*C*_0_ is the charging energy associated with the capacitance of each island to ground *C*_0_. This radiation damping impedance plays the role of *R*_damp_. Plasmon radiation damping is in stark contrast to radiation damping in normal electromagnetic transmission lines where the impedance scale is set by the free space impedance *Z*_0_. Electromagnetic transmission lines typically have impedance as ${Z}_{L}\simeq {Z}_{0}/2\pi =\tfrac{4}{\pi }{R}_{Q}\alpha$ where *α* = 1/137.036 is the fine structure constant. Thus $\sqrt{{E}_{C0}/8{E}_{J}}$ plays the role of the fine structure constant in the JJC.

When $\sqrt{{E}_{C0}/{E}_{J}}\gg 1$ (corresponding to $\alpha \gg 1$) and ${E}_{C}\mathop{ > }\limits_{ \tilde {}}{E}_{J}$, coherent QPS or large quantum fluctuations spanning many 2*π* windings of the phase, give rise to a classical description of the JJC dynamics in terms of well defined charge^20, 28^. This remarkable state of the JJC is a globally coherent state, something that we normally only associate with superconductivity, where the JJC is an insulator below a certain critical voltage. The charge dynamics in this state has a beautiful duality to phase dynamics in the parallel array of Josephson junctions, where transport is described in terms of charge solitons, the dual to flux solitons^5^. When the charge soliton length is larger than the JJC length, the array can be treated as a lumped element governed by dual relations to the DC and AC Josephson effects^20, 29, 30^. Achieving this dual state of a condensate of charged bosons in a circuit which can support a DC electrical current is of practical interest for quantum metrology. It is predicted that Bloch oscillations in such a circuit could be synchronized to an external signal^13, 31, 32^ resulting in a stable dynamical state where DC current is related to frequency via a fundamental constant, *I* = 2*ef*.

The ability to externally tune the parameters of a JJC is critical for experimental investigation, as many of these interesting phenomena are detectable only in a very narrow parameter range^10, 33, 34^. By fabricating the individual junctions of the chain in a loop geometry, each forming a Superconducting Quantum Interference Device (SQUID), we achieve tunability with an externally applied magnetic flux. If one link in the chain has a different loop area, we create a system with an independently tunable phase-slip center (PSC)^12^. In this way we form a single sample that can be tuned so that phase slips are either dominantly classical or dominantly quantum, and either localized to one spot in the chain or uniformly distributed throughout the chain.

In this paper we study DC transport in JJCs having a independently tunable, localized PSC in a chain of tunable Josephson junctions. For JJC’s with ${Z}_{A}\gg {R}_{Q}$ we observe a Coulomb blockade, the signature of coherent QPS in the chain. When ${Z}_{A}\ll {R}_{Q}$ our observations are well described in terms of nonlinear classical dynamics of the phase. We explain many features of the current-voltage characteristics (IVC) in this regime using a model which includes nonlinear damping at each junction. The simulation allows us to study the spatial and temporal distribution of classical phase-slips in the JJC, details which can not be measured in the experiments. The simulations show how classical phase slips at the PSC excite electromagnetic waves which propagate in the chain and reflect off the boundaries. At intermediate voltage bias, a coalescence of many reflected waves traveling in the nonlinear medium causes a temporal bunching of phase slips at the PSC. At higher bias, phase slipping at the weak link becomes more uniformly distributed in time. We also observe features in the IVC that are attributed to a gap in the linear mode spectrum, above the plasma frequency of the individual junctions in the JJC.

The paper is structured as follows: In section 2 we introduce our numerical model as well as the samples that were used in our experiments. In section 3 we present experimental results obtained for two different JJCs and we describe numerical simulations which reproduce the experimental IVC. Section 4 examines the simulations in terms of the spatial and temporal distribution of phase-slips. In the last section we summarise our results and conclude.

## Sample Design and Theoretical Model

**Figure 1:**
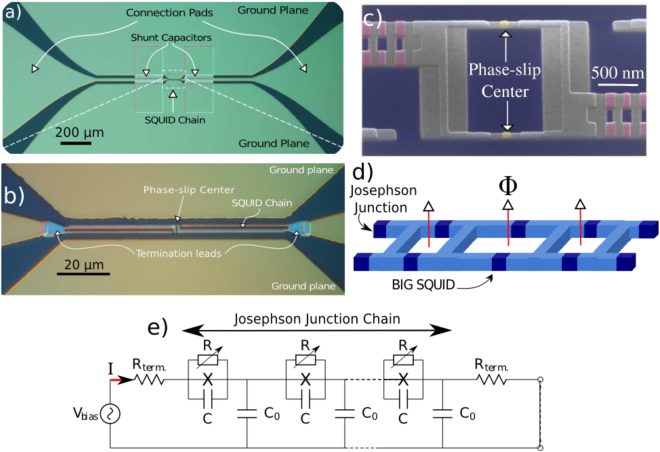
(**a**) Global Optical Microscope view of the Josephson junction chain named Sample 1. This sample has CPW geometry and the chain consists of 384 SQUIDs with total length of 100 *μ*m. Two thin-film capacitors with the sizes 200 *μ*m × 20 *μ*m are fabricated on chip and connected in series to shunt the chain. (**b**) Optical Microscope image of the center gap together with termination leads. (**c**) SEM image of the Josephson junction chain named “Sample 1”. The PSC *SQUID* visible at the center of the chain has smaller junction size (~2 × 95 nm×105 nm, coloured in yellow) and larger loop area (~12×) acting as phase-slip center. Chain SQUIDs (coloured in purple) with larger junction sizes (~2 × 100 nm × 300 nm) are located on either side of the PSC *SQUID*. SEM image is taken when the beam is tilted ~50° along with the direction of the arrows indicating the weak junctions of the PSC *SQUID*. (**d**) Sketch of the tunable-link chain named “Sample 2”. Different than Sample 1, the junction areas are the same for both chain SQUIDs and the tunable-link SQUID in this sample. But the loop area of the big SQUID is approximately twice as the chain SQUIDs. (**d**) Circuit model of the one-dimensional Josephson junction chain used for the simulations.

**Figure 2:**
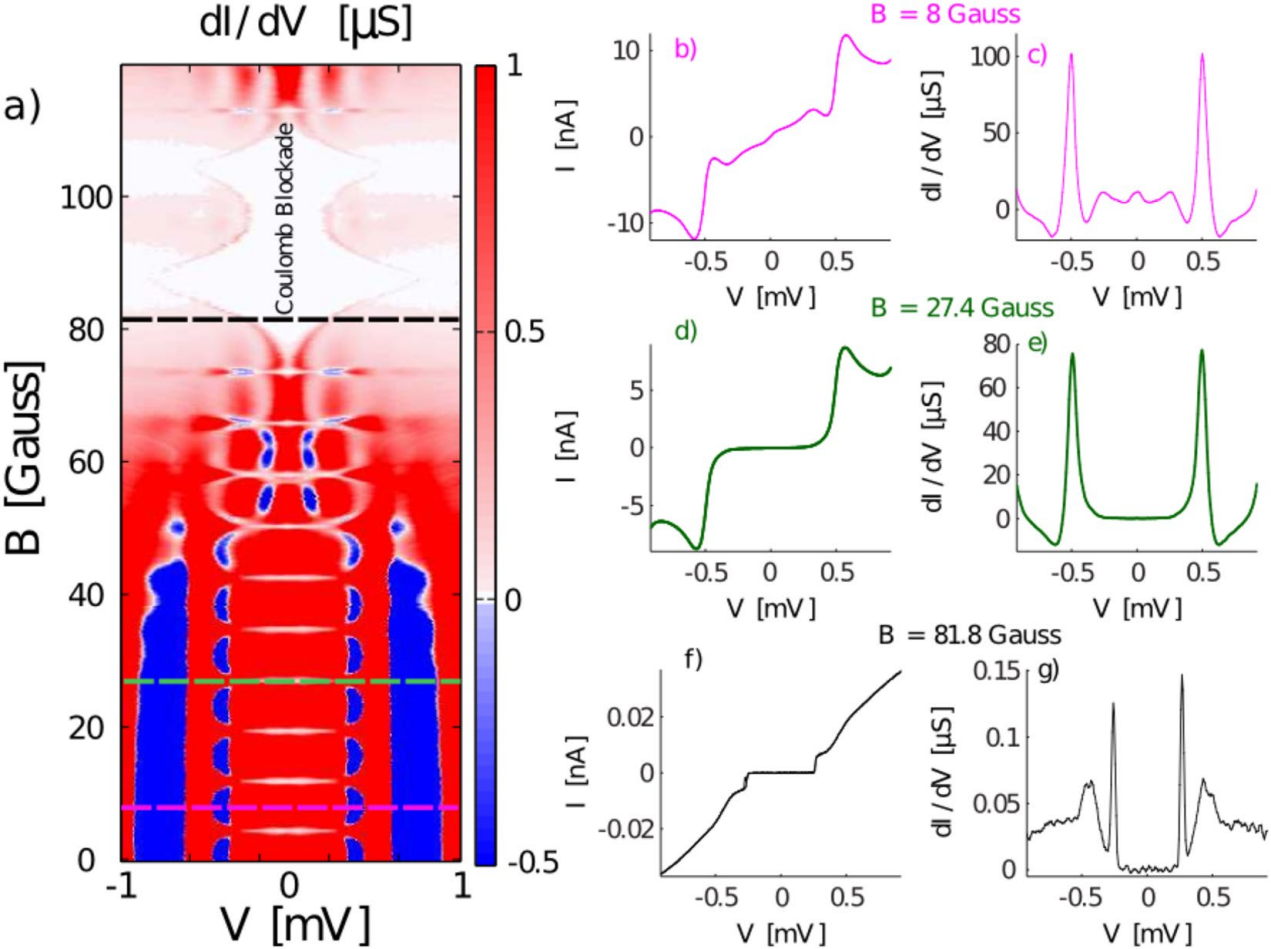
(**a**) Color map of the measured differential conductance of sample 1 (N = 384, *E*_*J*0_/*E*_*C*_ = 11.92 for the chain and ${E}_{J0}/{E}_{C}\simeq 1.33$ for the PSC) as a function of external magnetic field and bias voltage. For magnetic fields up to 50 Gauss the current-voltage characteristic is determined mainly by the PSC due to the localization of the phase-slips and the pattern with period 7.8 G corresponds to one flux quantum treading the PSC. At higher magnetic fields phase-slips in chain SQUIDs become important. The current-voltage characteristics at *B* = 81.8 *G* (f and g) shows a well-developed Coulomb blockade state with a distinct threshold voltage. The two maxima of the threshold voltage is due to two different areas of the SQUID loops in the chain. For magnetic field values when the weak-link is at the minimum, the dissipative branch above the threshold voltage shows a dip in the differential conductance due to the localization of the phase-slips at the tunable link.

The Josephson junction chains measured in this study consist of SQUIDs connected in series as shown in Fig. 1. We have designed and fabricated numerous chains of different length and tunnel junction resistance. In this paper we focus on two arrays with sample parameters given in Table 1, each containing a PSC. In both samples the chains are shunted by large capacitors (20 *μm* × 200 *μm*) located at each end of the chain. Sample 1 consists of 384 series SQUIDs (which we call chain SQUIDs) where one single SQUID has smaller junction size and larger loop area. This single SQUID is the PSC located exactly in the middle of the chain, as shown in Fig. 1a–c. Due to imperfections in the fabrication^35^ every other chain SQUID has a slightly different area. A consequence of this alternating area is a modulation of the transport properties with two different periods of magnetic flux. Hence, flux modulation results in two maximum of the threshold voltage in the Coulomb blockade regime, as seen in differential conductance plot of Fig. 2a. However, at lower flux bias, where the phase can be treated as a classical variable, the difference in coupling energy between alternating junctions is smaller, and we therefore model the array as uniform. Sample 2 has a slightly different geometry where all of the 2888 chain SQUIDS have nominally the same junction size. In this sample one single SQUID somewhere in the chain was defective, forming a loop area approximately twice that of all other SQUIDs (see Fig. 1d). This defect acted as a localized PSC, which could be verified by studying the flux tuning.

**Table 1:** Parameters of the Josephson Junctions chains mentioned in this paper: *N* is the number of series SQUIDs in the chain; *A*_*JJ*_ is the total area of two Josephson junctions in the loop which forms one SQUID in the chain; *A*_*L*_ is the total loop area of a SQUID formed by two parallel junctions; *R*_*N*_ = *R*_Tot_/*N* is the normal state resistance of each link in the chain, assuming a uniform chain; *E*_*J*0_ is the bare Josephson coupling energy of each link, *E*_*J*0_ = (*R*_*Q*_/*R*_*N*_)(Δ_0_/2), where Δ_0_ is the superconducting energy gap; *E*_*C*_ is the charging energy associated with the nearest neighbour capacitance *C*, determined from the area *A*_*JJ*_ and the specific capacitance *c*_*S*_ = 45 fF/*μ*m^2^, *E*_*C*_ = *e* ^2^/(2*c*_*S*_ *A*_*JJ*_); *E*_*J*0_/*E*_*C*_ is the ratio between two characteristic energies at zero external magnetic field; *f*_*p*_ = *w*_*p*_/2*π* is the estimated plasma frequency at zero external magnetic field.

Sample	*N*	*A*_*JJ*_/*μ*m^2^	*A*_*L*_/*μ*m^2^	*R*_*N*_/kΩ	*E*_*J*0_/*μ*eV	*E*_*C*_/*μ*eV	*E*_*J*0_/*EC*	*f*_*p*_/*GHz*
1 (Chain)	384	0.06	0.12	1.82	353.8	29.6	11.92	70
1 (PSC)	1	0.02	2.1	5.46	118	88.8	1.33	70
2 (Chain)	2888	0.05	0.12	0.48	1350	35.6	38	149
2 (PSC)	1	0.05	0.24	0.48	1350	35.6	38	149

The island stray capacitance is estimated to be *C*_0_ ≈ 15 aF.

Due to the SQUID design, the Josephson coupling energy *E*_*J*_ can be effectively tuned with an external magnetic field, *B*, threading the SQUID of loop area *A*_*L*_, as depicted in Fig. 1d.

1 $${E}_{J}=\frac{{R}_{Q}}{{R}_{{\rm{N}}}}\frac{{{\rm{\Delta }}}_{0}}{2}|\cos \,(\pi \frac{B{A}_{L}}{{{\rm{\Phi }}}_{0}})|$$

The difference in loop area between the chain SQUIDs and the PSC (see Table 1) enables tuning of Josephson energy more or less independently (Eq. 1). Sample 1 had a much larger area of the PSC and therefore we can modulate the coupling of the PSC from maximum to minimum with low magnetic field, without significantly altering the coupling in the chain SQUIDs (see Fig. 2a).

The circuit model used for numerical simulation is depicted in Fig. 1e. Each SQUID in the chain is modeled as an ideal Josephson junction shunted by a capacitance *C* and a nonlinear resistor *R*. The nonlinear resistor which models quasi-particle damping is infinite below the gap voltage *V*_*g*_ = 2Δ_0_/*e*, above which it switches discontinuously to the value *R*_*N*_. Damping is also supplied by resistors that terminate each end of the chain. These terminating resistors are a simple model of the leads to the JJC, treating them as semi-infinite microwave transmission lines with a frequency independent real impedance *R*_term_ = 50 Ω.

The dynamics in the chain is governed by the following system of equations,

2 $${\dot{\theta }}_{i}=\frac{2e{V}_{i}}{\hslash },$$

3 $${C}_{0}{\dot{V}}_{i}=-({I}_{i}^{{\rm{tot}}}-{I}_{i-1}^{{\rm{tot}}}),$$

where *θ*_*i*_ is the phase of the superconducting order parameter at island *i*, *V*_*i*_ is the voltage, and

4 $${I}_{i}^{{\rm{tot}}}={I}_{c,i}\,\sin \,({\theta }_{i}-{\theta }_{i+1})+C({\dot{V}}_{i}-{\dot{V}}_{i+1})+{I}_{i}^{R}$$

is the total current flowing through the junctions between island *i* to *i* + 1, for 0 < *i* < *N*. The quasiparticle current is modeled as

5 $${I}_{i}^{R}=\{\begin{array}{ll}({V}_{i}-{V}_{i+1})/{R}_{N}+{I}_{i}^{n} & {\rm{if}}\,|{V}_{i}-{V}_{i+1}| > {V}_{g}\\ 0 & {\rm{otherwise}}\end{array},$$

where *R*_*N*_ is the normal resistance of a single junction. (The subgap resistance is thus assumed to be infinite). The currents entering the chain from the left *I*_0_ and leaving from the right *I*_*N*_ via the terminating resistors are

6 $${I}_{0}^{{\rm{tot}}}=(U-{V}_{1})/{R}_{{\rm{term}}}+{I}_{0}^{n},\quad {I}_{N}^{{\rm{tot}}}={V}_{N}/{R}_{{\rm{term}}}+{I}_{N}^{n},$$

respectively, where U is the applied bias voltage, and ${I}_{i}^{n}$ are Gaussian Johnson-Nyquist noise currents with zero mean and autocorrelation $\langle {I}_{i}^{n}(t){I}_{j}^{n}(t^{\prime} )\rangle =\tfrac{2{k}_{B}T}{R}{\delta }_{ij}\delta (t-t^{\prime} )$. From the time series of the simulation one may identify the phase slips as events where the phase difference *θ*_*i*_ − *θ*_*i*+1_ crosses the discrete values (2*m* + 1)*π* for integer *m*.

Simulation consists of numerical integration of the coupled set of nonlinear differential equations describing the phase dynamics of the chain. During the simulation we track the time and position of phase slip event. Averaging over many events, we extract the average current at fixed voltage bias. The simulation includes only thermal fluctuations, whereas in the experiment we surely have both thermal and quantum fluctuations. Due to the relatively low impedance of the terminating resistors the arrays are essentially voltage biased, which means that the total rate of phase slips between the two ends of the chain is fixed by the applied voltage. Below we describe simulations which reproduce many detailed features of the experimental IVC, allowing us to examine the spatial and temporal distribution of phase slips. Further details of this model are presented in ref. 36.

## Results

We measured the current-voltage characteristics of the chains while the external magnetic field was increased step-wise. Figure 2a shows a color map of the differential conductance of sample 1 as a function of bias voltage and magnetic field. In Fig. 2b–f the IVC and differential conductance are shown at three different field values marked by dashed lines of the corresponding color in Fig. 2a. We distinguish between a low field regime below ~50 G and a high field regime above ~70 G.

In the low field regime we observe a pattern with a period of 7.8 *G* corresponding to one flux quantum threading the PSC. The field modulation shows that transport in this regime is dominated by phase-slips at the PSC. The differential conductance at maximum *E*_*J*_ of the PSC (Fig. 2c) shows a zero bias peak, as well as peaks at finite voltage bias. When the bias voltage reaches the superconducting gap voltage, $2{{\rm{\Delta }}}_{0}/e\sim 0.4\,mV$, a step in the IVC and large peak in the differential conductance are observed, indicating that Cooper pair tunnelling at finite voltage is greatly enhanced by dissipative quasi-particle tunnelling. Below and above the gap voltage, regions of negative differential conductance are observed. Also in this low-field regime we show the differential conductance at minimum *E*_*J*_ of the PSC (Fig. 2d). Here the zero bias peak as well as the other peaks below the gap voltage are suppressed, and no negative differential conductance is observed below the gap voltage.

The high-field regime for magnetic fields above ~70 *G* is labeled Coulomb blockade in Fig. 2a. In this regime it is the phase-slips in the chain which dominate and the IVC shows a well-developed Coulomb blockade state with a distinct threshold voltage (Fig. 2f) similar to that observed in uniform chains^12^. The threshold voltage is clearly modulated with flux in the chain SQUIDS, leading to two maxima at 90 *G* and 105 *G*, corresponding to the two different SQUID areas. The peaks in differential conductance at the gap voltage are strongly suppressed and new peaks at the threshold voltage are observed (Fig. 2f).

**Figure 3:**
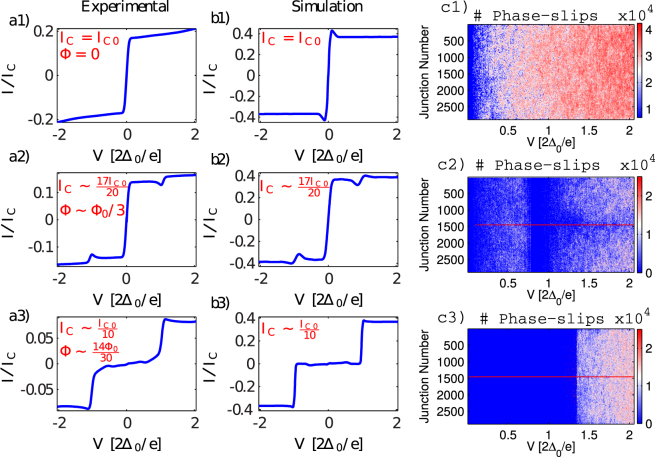
Experimental (**a1**–**a3**) and simulated (**b1**–**b3**) dc I–V curves together with the color plot of the phase-slips (**c1**–**c3**) for various magnetic field and *E*_*J*_ values. The criteria for describing the location of the phase-slips is specified in the “*Sample design and theoretical model*” section.

Sample 2 is a much longer chain (2888 SQUIDs in series) and the PSC has a loop area only twice that of the chain SQUIDs. Hence the magnetic field modulation has larger period for sample 2 in comparison to sample 1. Nevertheless, many features of the IVC and differential conductance are common to both samples. In Fig. 3 we compare the simulated and measured IVC of Sample 2 for three different values of the magnetic flux threading the PSC. At zero magnetic field all of the SQUIDS in the chain (including the PSC) have the same Josephson energy and the chain is homogeneous. The measured (Fig. 3a1) and simulated (Fig. 3b1) IVCs consist of a supercurrent-like branch with some finite resistance (i.e. not a pure zero-voltage current). At higher bias the supercurrent-like branch gives way to a constant-current branch. Previously we have shown how this constant-current branch is the result of random phase-slip and phase-stick events uniformly distributed throughout the chain^36^. This uniform distribution can be seen in the color density plot showing the total number of phase slips during the simulation, in the plane of junction number and bias voltage (Fig. 3c1).

As the magnetic field is increased from zero, the flux threading the PSC will be twice that of the chain SQUIDs. Hence the PSC has lower *E*_*J*_ and therefore a lower energy barrier for phase-slips. At a flux bias of 0.33Φ_0_ we observe a dip in the IVC for voltages around 2Δ_0_/*e* as shown in Fig. 3a2,b2. Figure 3c2 show that the phase-slips become concentrated at the PSC in the center of the chain, however with some residual phase-slips occuring in the rest of the chain. The behavior of the chain changes drastically when the magnetic flux through the PSC is close to the half flux quantum, Φ = 0.45Φ_0_ when the PSC is at a minimum of *E*_*J*_. Figure 3a3,b3 show that the current is drastically suppressed for the bias voltages up to the gap voltage. Furthermore, Fig. 3c3 shows that the phase slips in this low-bias region are entirely concentrated at the PSC. In this region the rest of the array essentially acts as a high impedance environment^9^.

**Figure 4:**
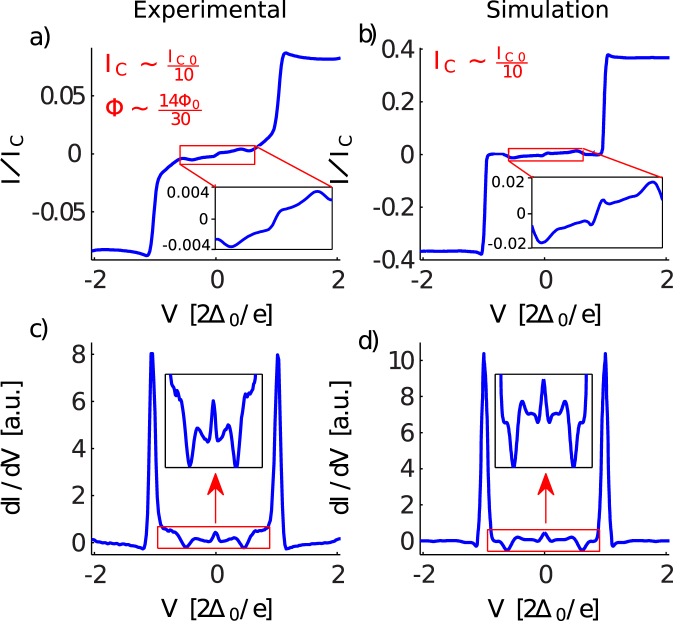
Experimental dc I–V curve (**a**) and differential conductance (**c**) of the Sample 2 as a function of bias voltage for the external magnetic flux, Φ = 14Φ_0_/30 and the critical current *I*_*C*_ = *I*_*C*0_/10. Simulated dc I–V curve (**b**) and differential conductance (**d**) for the same sample. Insets show the zoom the to low bias voltage intervals.

The remarkable correspondence between measured and simulated IVCs can be seen in Fig. 4 where we plot the IVC as well as the differential conductance of sample 2 at magnetic flux Φ = 0.45Φ_0_ where phase slips are concentrated at the PSC. From this comparison we conclude that our model and simulation capture the essential features of the phase-dynamics in the chains. We emphasize that all the parameters used the simulation are either experimentally measured or estimated from sample geometry. There are no free fitting parameters. The parameters not listed in Table 1 are: the thermal noise level *k*_*B*_ *T*/*E*_*J*_ = 0.001 and capacitance to the ground *C*_0_/*C* = 0.009.

## Discussion

### Dynamics at the phase-slip center

**Figure 5:**
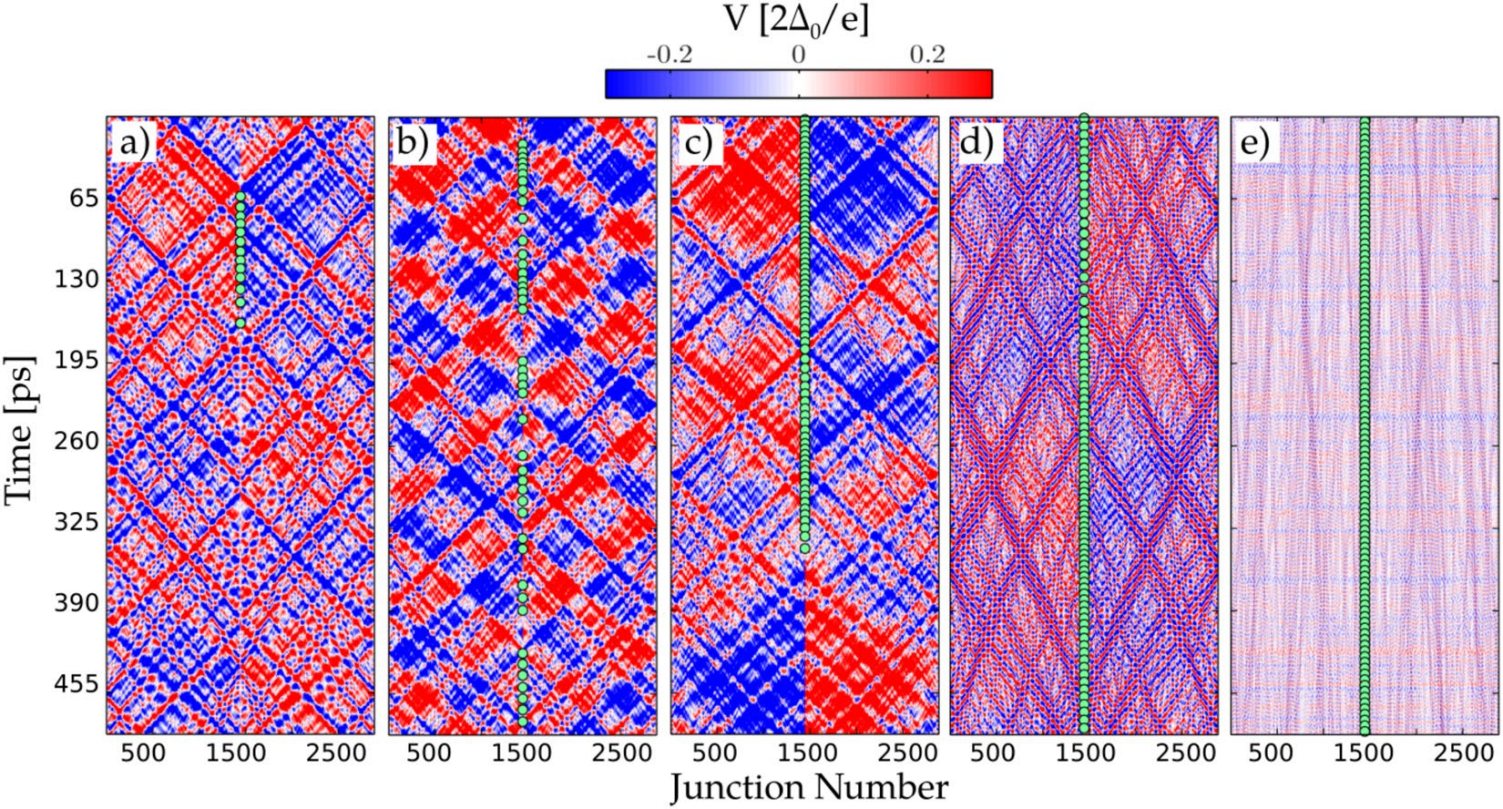
Color plot of the voltage profile and the phase-slip distribution across the JJC for various bias voltages, *V* = 0.1,0.3,0.5,0.7,0.9 $[2{{\rm{\Delta }}}_{0}/e]$, from (**a**–**e**) accordingly. The IV characteristics is shown in Fig. 4b. Each green circle represents a phase-slip event and all of them are localized at the PSC junction. Simulations show different voltage propagation modes exist throughout the JJ chain for different bias voltages.

Simulation also allows us to track the temporal location of phase slip events which we describe in this section. We concentrate on the case where the central junction has weak enough coupling, such that all phase slips are located at the PSC. Figure 5 shows color plots of the time evolution of voltage at each island in the JJC, or the potential difference between each island and ground, $V(x,\,t)=\tfrac{\hslash }{2e}\dot{\theta }(x,\,t)$. The parameters used in the simulations corresponds to IVCs displayed in Fig. 4b and the differential conductance displayed in Fig. 4d.

Each panel of Fig. 5 corresponds to a different voltage bias, or net phase slip rate, with panel a) being lower bias and panel e) being higher bias. Phase slip events are plotted as green circles in each panel, occurring only at the PSC. One can see diagonal features which correspond to voltage pulses propagating in the array. A phase slip event at the PSC gives rise to a positive (red) and negative (blue) voltage pulse on opposite sides of the PSC. These propagate outward with a velocity $v\sim 1/\sqrt{{L}_{J}{C}_{0}}=\sqrt{8{E}_{J}{E}_{C0}}/\hslash \approx 14$ junctions/ps toward the edges of the array where they are reflected, changing sign upon reflection. This type of reflection occurs because the terminating resistance is much lower than the transmission line impedance of the array. In other words, the density of modes for the distributed Josephon plasmons in the JJC is much larger than that of electromagnetic modes in the leads^25, 37, 38^.

In Fig. 5 one can see that at higher bias voltage, just below the gap voltage, phase slip events become more uniform in time, eventually settling in to periodic behavior (Fig. 5e). In this high bias regime the diagonal features become washed out, but remnants of the traveling voltage pulses are still seen, however now traveling at half the velocity (Fig. 5d). In contrast, at low bias the phase slip events tend to bunch in time. This may be explained by nonlinearity and dispersion of the traveling wave components of the voltage pulses. Apparently the phase of these propagating waves tends to coalesce, resulting in large potential gradients which enhance phase-slipping.

**Figure 6:**
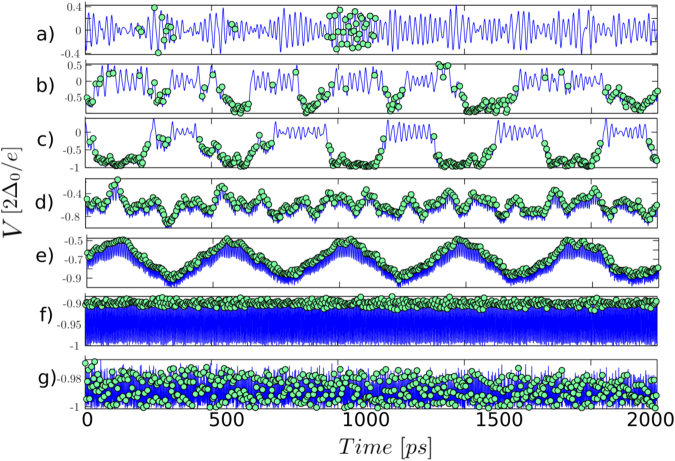
Voltage oscillations and phase slip distribution at the PSC for various bias voltages, V = 0.05, 0.25, 0.45, 0.65, 0.75, 0.95, 1.01 $[2{{\rm{\Delta }}}_{0}/e]$, from top to bottom. Different oscillation patterns and frequencies are observed and generated by the nonlinear response to phase slips. The highest oscillation frequency observed is ~200 GHz, corresponding to f = 4 Δ_0_/*h*.

A detail of the potential gradient, or voltage drop across the PSC versus time is shown Fig. 6. Here we again see the temporal bunching (Fig. 6a–c), giving way to a constant phase slipping rate (Fig. 6f,g) which is fixed by the external voltage bias. At intermediate bias voltage, we see that the voltage drop across the PSC begins to oscillate in time (Fig. 6d,e), indicating that the phase slip rate itself settles in to periodic oscillations.

**Figure 7:**
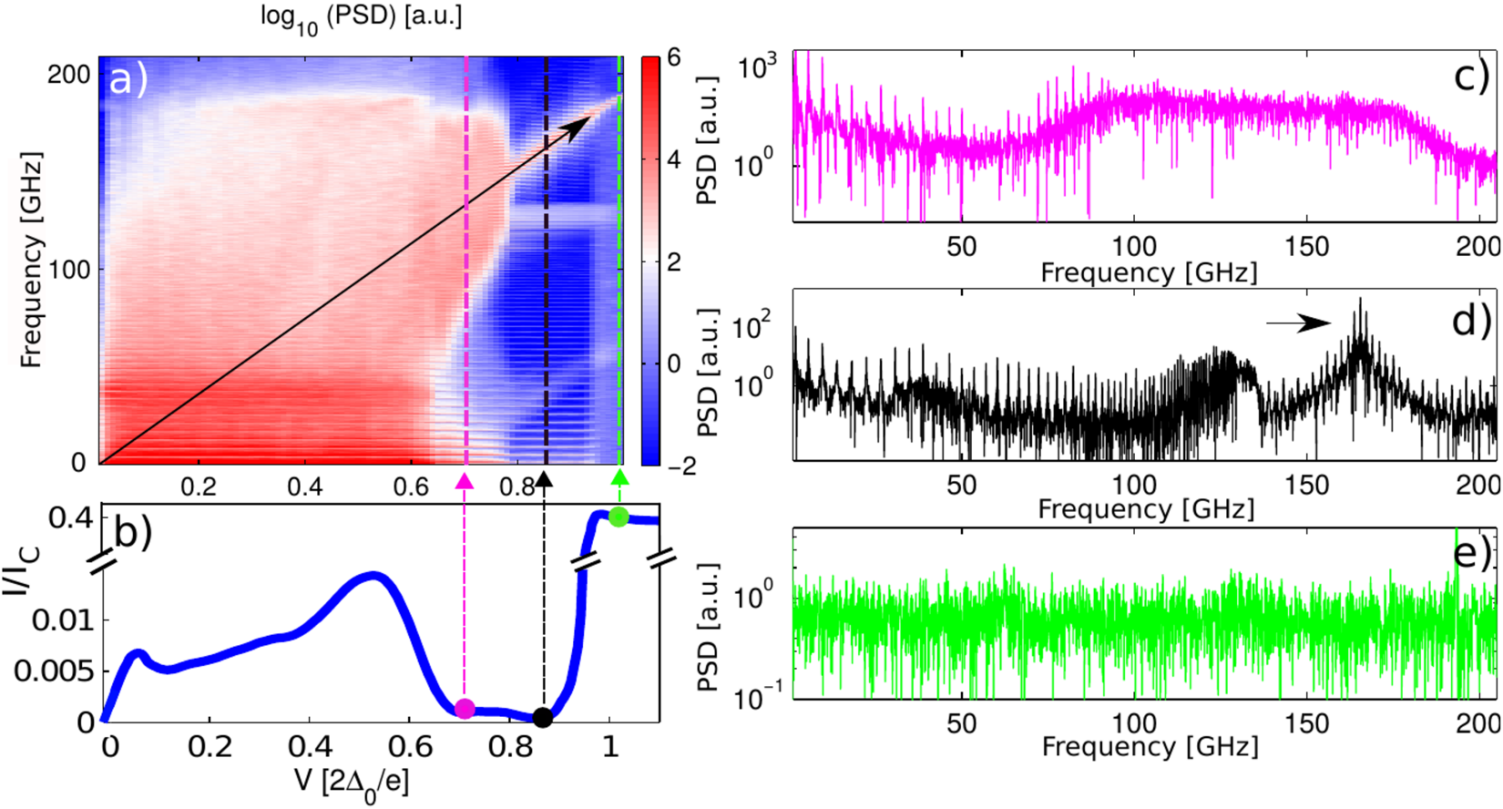
(**a**) Power Spectral Density (PSD) of the voltage signal created by the PSC as a function of bias voltage and frequency. The black diagonal arrow indicates the frequency of the Josephson oscillations as a function of bias voltage, *f* = 2*eV*_bias_/*h*. (**b**) Simulated dc-IV curve with the three bias points (magenta, black and green) representing the dashed lines in (figure a). (**c**) PSD profile for the bias voltage $V=0.7\,[2{{\rm{\Delta }}}_{0}/e]$. Frequency of the Josephson oscillations at this voltage bias is similar to the plasma frequency of the PSC, *f*_*p*_ ~ 149 GHz. (**d**) PSD profile for the bias voltage $V=0.85\,[2{{\rm{\Delta }}}_{0}/e]$. Low frequency behavior of the PSD shows discrete plasmon modes and the accumulation of the frequency peaks up to the plasma frequency. The Josephson oscillation peak corresponding to the applied voltage is also visible and marked with an arrow. (**e**) PSD profile for a bias voltage above the superconducting gap, $V=1.02\,[2{{\rm{\Delta }}}_{0}/e]$.

To further understand the complex dynamics in the JJC, we calculate the power spectrum of the signal corresponding to the voltage drop across PSC (Fig. 6). Figure 7a shows a color plot of the signal power, calculated by squaring the Fourier transform of the simulated time series, in the plane of frequency and bias voltage. One can clearly see an abrupt drop in signal power for frequencies above ~190 GHz. This frequency corresponds the Josephson oscillation frequency of the individual junctions, for a bias corresponding to the superconducting gap of the junction, ${V}_{{\rm{bias}}}=[2{{\rm{\Delta }}}_{0}/e]$.

Figure 7b shows the simulated IV curve for the same voltage bias range as in Fig. 7a. A maximum of the sub-gap current appears around $V=0.55\,[2{{\rm{\Delta }}}_{0}/e]$ which represents the Josphson oscillations driven by the applied bias becoming comparable to the superconducting gap, $f=2{{\rm{\Delta }}}_{0}/h\sim 100\,{\rm{GHz}}$. The microwaves emitted by the Josephson oscillations at the PCS may in principle excite quasiparticles if the frequency exceeds the superconducting gap, thereby causing a nonequilibrium density of quasiparticles in the chain. This is not accounted for in the simulations. However, for our experimental samples the effect seems small, since in the IV-characteristic we observe states with zero or almost zero current which indicates that no power is dissipated (Fig. 4a).

This maximum shown in Fig. 7b is followed by a region with a negative slope and a drastic suppression of the current level. The suppression of the current and the signal power stretches between the bias voltages $0.7\,[2{{\rm{\Delta }}}_{0}/e] < {V}_{{\rm{bias}}} < 0.95\,[2{{\rm{\Delta }}}_{0}/e]$. The low-bias edge of this region corresponds to the Josephson oscillations becoming equal to the plasma frequency, or cutoff in the plasmon spectrum of the JJC, $2e{V}_{{\rm{bias}}}/h\sim {f}_{p}$ ^25, 37, 38^. We therefore explain this suppression of current as being the result of the gap in the plasmon mode spectrum: Single Cooper pair tunneling at finite voltage is suppressed because there are no plasmon modes that can be excited to dissipate the energy 2*eV*_bias_ supplied by the source.

We can see this plasmon spectrum gap directly in the PSD if we make a cut at bias voltage ${V}_{{\rm{bias}}}=0.85\,[2{{\rm{\Delta }}}_{0}/e]$ (black dashed line in Fig. 7a). For this bias voltage we observe a minimum of the current. For frequencies lower than the plasma frequency the power spectrum shows the discrete plasmon modes of the finite-length JJC (Fig. 7d). A linear analysis of a homogeneous JJC shows that the frequencies of these modes follow the dispersion relation^37, 38^,

7 $${\omega }_{k}={\omega }_{p}\sqrt{\tfrac{1-\,\cos \,k}{1-\,\cos \,k+{C}_{0}/2C}}$$

where *k* = *nπ*/*N* is the mode number. The modes become more dense with increasing frequency until the gap edge at the plasma frequency. Interestingly, our simulation of the full nonlinear dynamics does show a peak in the signal power in this gapped region of the spectrum, when the Josephson oscillation frequency *f* = 2*eV*/*h* corresponds to the applied voltage (peak marked with an arrow in Fig. 7d).

Furthermore, the position of the two maximum points corresponding to ${V}_{{\rm{bias}}}=[{{\rm{\Delta }}}_{0}/e]$ and ${V}_{{\rm{bias}}}=[2{{\rm{\Delta }}}_{0}/e]$, does not change as the external magnetic field tunes the Josephson coupling. However the minimum point, which corresponds Josephson oscillations becoming equal to the plasma frequency, does show the expected oscillation with the flux threading the SQUID loop area (Fig. 2a).

We describe experimental measurements and simulations on two Josephson junction chains with a separately tunable phase-slip center. The experimental current voltage characteristics display many features at bias voltages lower than the gap voltage that we reproduce by simulating the nonlinear dynamics. Simulations were performed on a model where damping was provided by nonlinear quais-particle resistance shunting each junction, as well as finite terminating resistance. Our model accounts only for the classical phase-slips and therefore it could not reproduce Coulomb blockade behavior observed in the experiments when the array impedance exceeds *R*_*Q*_. Simulations show how phase-slips launch traveling voltage pulses which reflect at the array edges. These pulses tend to coalesce and trigger new phase-slip events which become bunched in time. By analyzing the power spectrum of voltage oscillations at the PSC, we could see how the density of distributed plasmon modes affect the IVC of the chain.

## Methods

Josephson Junction chains have been fabricated with an electron beam lithography system by stitching several 100 *μ*m wide write fields together. Electron beam lithography system was equipped with a laser interferometer stage and also with automatic alignment functionality and therefore the stitching errors were minimal. In order to provide filtering, on chip shunt capacitances are fabricated close to the each termination of the chain with the distance of ~120 *μ*m. *Al*/*Al*_2_ *O*_3_ shunt capacitors are defined by optical lithography on *Si*/*SiO*_2_ substrate. Au ground planes are deposited in an UHV deposition system with PVD technique. The electron beam masks for the Josephson junction chains were patterned with the electron beam lithography system. Two-layer resist process, PMGI-SF7 & Zep520A, is used with over-development of the bottom layer to obtain large undercut and free-standing bridges. The junctions were formed by two-angle *Al* evaporation with *in situ* oxidation to form overlapping *Al* islands with an *Al*_2_ *O*_3_ tunnel barrier.

Low-temperature measurement were carried out in an Oxford 3He/4He-dilution refrigerator with a base temperature of ~15 mK. A standard two-terminal configuration is used for the current-voltage measurements and the cryogenic system was equipped with a magnet. A magnetic field perpendicularly to the substrate plane of the devices was applied in order to tune the critical current value of the Josephson junction chains.

## Electronic supplementary material

Supplementary Video for the Experimental DCIV

Supplementary Video for the Simulated Voltage Oscillations

Explanations of the Supplementary Video Files
