# Supplementary material for: Spatial and temporal distribution of phase slips in Josephson junction chains
Source: Sci Rep. 2017 Sep 13;7:11447. doi: 10.1038/s41598-017-11670-7 (PMC5597633; doi:10.1038/s41598-017-11670-7)
Supplement: Supplementary file 3 — Explanations of the Supplementary Video Files [file 41598_2017_11670_MOESM3_ESM.pdf]

Spatial and temporal distribution of phase slips in Josephson junction chains

Adem Ergül[1,*,+], Thomas Weißl[1], Jan Johansson[2], Jack Lidmar[3], David B. Haviland[1]

[1]Nanostructure Physics, Royal Institute of Technology, SE-106 91 Stockholm, Sweden
[2]Department of Natural Sciences, University of Agder, Kristiansand, Norway
[3]Theoretical Physics, Royal Institute of Technology, SE-106 91 Stockholm, Sweden
[*]adem@kth.se
[+]Current address: Department of Physics, Stockholm University, SE-106 91 Stockholm, Sweden

Supplementary Materials

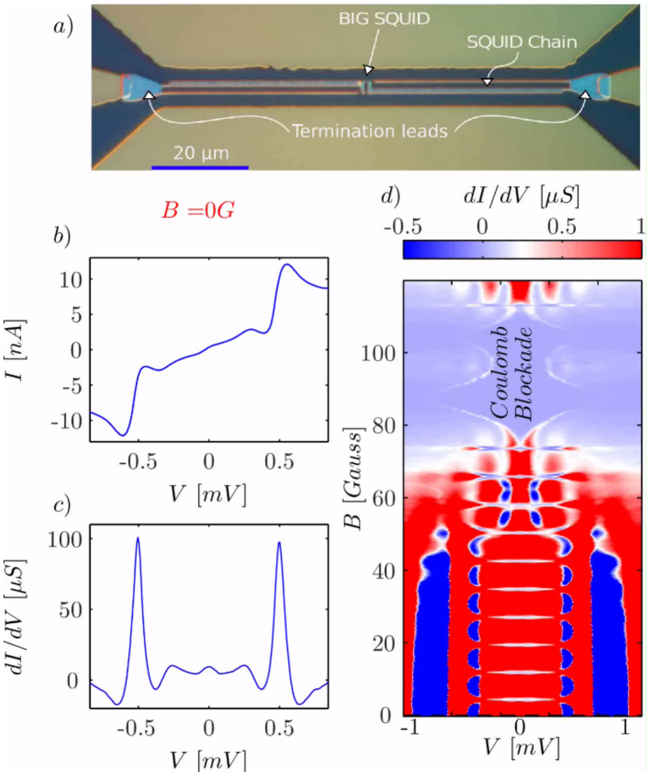

Figure 1. Experimental current-voltage characteristics of the Josephson junction chain called "Sample 1" as a function of external magnetic field. First frame of the Supplementary Movie 1 is shown in this figure.

Movie 1 - Critical current tuning of Sample 1 with an external magnetic field

Movie 1 shows the current-voltage characteristics and the differential conductance of Sample 1 as a function an external magnetic field which is tuned from 0 to 120 Gauss in steps of 0.2 Gauss. Sub-figure "a" shows the Optical Microscope image of the Sample 1. Sub-figure "b" shows the experimental current-voltage characteristics (DC-IV) and sub-figure "c" shows the differential conductance (dI/dV) as a function of bias current. Sub-figure "d" shows the color map of the measured differential conductance of the Sample 1 as a function of external magnetic field and bias voltage. The y-scale of the sub figures "b" and "c" are adjusted at the external magnetic field values $B = 60, 80, 110$ Gauss values in order to show the details of the plots.

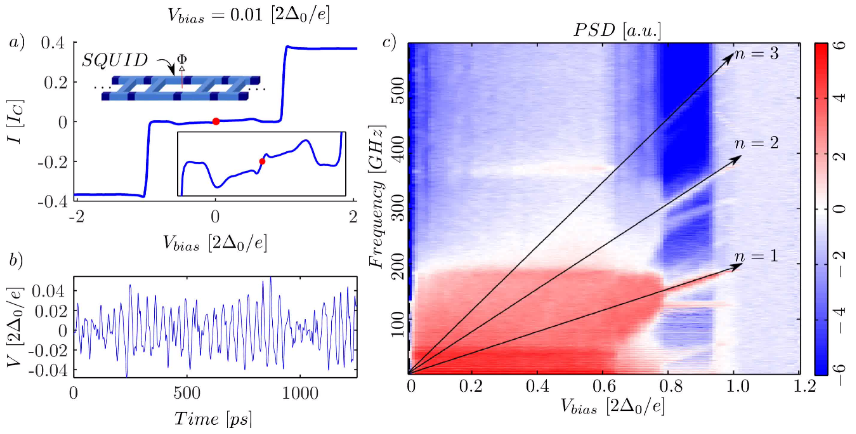

Figure 2. Simulated phase dynamics of the PSC junction embedded in the Josephson junction chain called "Sample 2" for the voltage bias values from 0.01 up to the 1.1 with the steps of 0.01 $[2\Delta_0/e]$. First frame of the Supplementary Movie 2 is shown in this figure.

Movie 2 - Simulated phase dynamics of the PSC junction embedded in Sample 2

Movie 2 presents the simulated phase dynamics of the PSC junction embedded in the Sample 2 for the bias voltage values starting from 0.01 up to the 1.1 $[2\Delta_0/e]$ with the steps of 0.01 $[2\Delta_0/e]$. Sub-figure "a" shows the simulated current-voltage characteristics (DC-IV curve) for the external magnetic flux value, $\Phi = 14\Phi_0/30$ and the critical current value, $I_C = I_{C0}/10$. Red dot on the DC-IV curve represents the point in which the phase dynamics is analyzed. Upper inset of this figure shows the sketch of the JJC and lower inset shows the zoom the to low bias voltage interval. Sub-figure "b" shows the simulated voltage fluctuations/oscillations at the PSC junction as a function of time and sub-figure "c" shows the Power Spectral Density of the voltage signal created by the PSC junction as a function of bias voltage and frequency.

The simulations successfully reproduced all the observed features in the current voltage characteristics. Due to the big difference in the critical current value of the chain SQUIDs and the PSC, $I_C = I_{C0}/10$, it is safe to argue that all the phase-slips and therefore dissipation is localized to the PSC (weak SQUID) and JJ chain acts only as a high impedance environment. Therefore phase dynamics of the PSC defines the current voltage characteristics of the whole chain for the bias values, $V_{bias} < |2\Delta_0/e|$. PSD analysis shows that the behavior of the PSC is strongly depends on bias voltage value and the simulated IVC consists of four distinct regions. In the first part, $0 < V_{bias} < 0.06$ $[2\Delta_0/e]$, the IVC shows a supercurrent branch. In this region, we do not see a significant dissipation and all the junctions of the chain including the PSC shows supercurrent behavior. The second region stretches between $0.06 < V_{bias} < 0.65$ $[2\Delta_0/e]$ and the PSC shows voltage oscillations around zero bias. These semi-periodic oscillations are interrupted when a phase-slip event happens due to the 2π unwinding of the phase. As the bias voltage increased, total number of phase-slips increases therefore the switching between these two modes become more frequent. And finally, when the bias value reaches to the $V_{bias} = 0.65$ $[2\Delta_0/e]$, voltage drop across the PSC

changes drastically and we start to see two beating modes of oscillations. Furthermore, this change emphasis itself as a dip feature both in IVC and differential conductance. Frequency of these beating oscillations increases as the bias voltage increased. Finally when the bias current reaches to the gap value, $V_{bias} \sim 2\Delta_0/e$, the PSC switches completely to the normal conducting state causing a steep jump on the current voltage characteristics. For the bias values larger than the gap value, the thermal noise dominates the voltage behavior of the PSC and phase-slips become totally random.

Audio File

A Josephson Junctions can be though as an ideal dc/ac converter, i.e. as a perfect voltage to frequency converter. When a Josephson Junction biased with a finite voltage, V_{bias}, the phase difference across the junction increases linearly by time and the supercurrent across the junction oscillates with a certain frequency called Josephson frequency;

$$f_J = \frac{2e}{h}\ V_{bias}$$

Using the simulated voltage difference values across the PSC, we calculate the frequency of Josephson oscillation at the PSC. For this estimation we have used the Josephson constant;

$$\Phi_0 = \frac{h}{2e} \sim 483\ \mathrm{THz/V}$$

After calculating the Josephson oscillation frequencies ($\sim$ 200 GHz) happening at the PSC, we have down converted these values to the audible frequency range ($20 - 20,000$ Hz) and created an audio file. At the final step, we overlay this audio file with the video showing the simulated results and created a sound motion picture to present the Josephson oscillations happening at the PSC both visually and auditorily.
